# Aptamers in Education: Undergraduates Make Aptamers and Acquire 21st Century Skills Along the Way

**DOI:** 10.3390/s19153270

**Published:** 2019-07-25

**Authors:** Gwendolyn M. Stovall, Vincent Huynh, Shelly Engelman, Andrew D. Ellington

**Affiliations:** 1 Texas Institute for Discovery Education in Science, Freshman Research Initiative, University of Texas at Austin, Austin, TX 78712, USA; 2 Texas Institute for Discovery Education in Science, High School Research Initiative, University of Texas at Austin, Austin, TX 78712, USA; 3 Texas Institute for Discovery Education in Science, University of Texas at Austin, Austin, TX 78712, USA; 4 Institute for Cellular and Molecular Biology, University of Texas at Austin, Austin, TX 78712, USA

**Keywords:** aptamer, calf-intestinal alkaline phosphatase, course-based undergraduate research experience, in vitro selection, Systematic Evolution of Ligands by Exponential Enrichment, SELEX, CURE, Freshman Research Initiative, 21st century skills

## Abstract

Aptamers have a well-earned place in therapeutic, diagnostic, and sensor applications, and we now show that they provide an excellent foundation for education, as well. Within the context of the Freshman Research Initiative (FRI) at The University of Texas at Austin, students have used aptamer selection and development technologies in a teaching laboratory to build technical and 21st century skills appropriate for research scientists. One of the unique aspects of this course-based undergraduate research experience is that students develop and execute their own projects, taking ownership of their experience in what would otherwise be a traditional teaching lab setting. Of the many successes, this work includes the isolation and characterization of novel calf intestinal alkaline phosphatase (anti-CIAP) RNA aptamers by an undergraduate researcher. Further, preliminary survey data suggest that students who participate in the aptamer research experience express significant gains in their self-efficacy to conduct research, and their perceived ability to communicate scientific results, as well as organize and interpret data. This work describes, for the first time, the use of aptamers in an educational setting, highlights the positive student outcomes of the aptamer research experience, and presents the research findings relative to the novel anti-CIAP aptamer.

## 1. Introduction

With a 29-year history, aptamers have a well-established presence in diagnostic, therapeutics, and sensor technologies [1,2,3]. Furthermore, with time the range of aptamer applications has broadened, with some applications stretching the field in interesting ways, including their use as molecular recognition elements (e.g., ELISA/ELONA, [4]), imaging elements (e.g., within live cells, [5]), DNA origami and nanorobots [6], and now aptamers have taken a foothold in education. As students increasingly choose to explore engineering approaches to biology, often lumped under the mantle of “synthetic biology” an educational introduction to aptamers provides one of the best means for fomenting their interest and providing them with the skills needed for professional success.

Aptamer technological and scientific advancements have been adopted and adapted to long-term course-based undergraduate research experiences (CURE). Meeting the urgent call for authentic research experiences in education [7,8,9], CURES have emerged throughout STEM fields. For example, with the emergence of smart phone technologies and sensor peripherals, a CURE devoted to DIY Diagnostics was developed (University of Texas, UT-Austin [10]). Similarly, with the advent of data analytics, the University of Maryland’s Sustainability Analytics CURE was established [11]. Likewise, CUREs using CRISPR (UT-Austin, [10]), nano chemistry (UT-Austin, [10]), biomaterial design (Iowa State University, [12]), as well as other technologies and advancements have emerged across the country (see “Replication Sites,” [10]). 

Critical to these authentic research experiences for undergraduates is the institutional support of undergraduate research programs, such as through the Freshman Research Initiative (FRI, UT-Austin, UT Rio Grande Valley, and Iowa State University), the First-year Innovation & Research Experience (FIRE, University of Maryland), Program to Educate and Retain Students in STEMS Tracks (PERSIST, UT El Paso), and the Achieving Success through Undergraduate Research and Engagement (ASSURE, UT Arlington) (programs listed in [10]). 

UT-Austin launched the FRI in 2006 with three CUREs. An aptamer selecting CURE, named the Aptamer Stream, was among these original teaching research labs. As of 2019, in its thirteenth year, the FRI includes 29 different CUREs offering authentic research experiences across a variety of disciplines and research areas, including robots, nanomaterials, computer security, organic chemistry, molecular biosciences, and more. Approximately 900 freshman students at The University of Texas participate in this program annually, with 50% of the students from underrepresented minorities [10]. 

Central to CURES and the supporting undergraduate research programs are the undergraduate students and the desire to positively impact and retain them over time. Previous outcomes of such initiatives have shown that participation in CURES leads to a 17% increased college graduate rate in 6 years; a 23% increased likelihood of earning a science, engineering, or mathematics degree (UT-Austin FRI, [13]); increased student interest in science, college retention, and science course grade (SEA Phage Hunters Program, [14]); increased independence and knowledge gains [15]; and, increased confidence and understanding of the research process [16,17], and, in the case of the UT-Austin FRI students, an estimated 16% more in lifetime earnings [18]. 

Through the UT-Austin FRI (briefly described in [19]), the Aptamer Stream students participate in a yearlong, two-semester research experience (i.e., spring to fall with an optional summer session). Freshman and sophomore students perform aptamer research under the guidance of a non-tenure track faculty member (co-author Stovall, G.M.), a tenure-track principal investigator (co-author Ellington, A.D.), and near-peer mentors. In the first spring semester, approximately 35 freshman students perform *in vitro* aptamer selections in small groups using parallel methodology. While receiving lower-division chemistry lab course credit, students begin their technical training in the beginning of the first semester, performing an aptamer selection (such as against egg-white lysozyme). In the latter half of the first semester, students, working individually, begin a new aptamer selection against one of the provided targets. Students have the option to continue their research over the summer, where they may progress on their aptamer selections and assay the selected pools for sequence enrichment (i.e., sequencing) and/or binding. During this fall semester, students receive lower-division biology course credit, continue their aptamer selection, assay their selected pool, characterize aptamers, and/or develop an aptamer application. The traditional research course sequence concludes in the fall semester of students’ sophomore year.

Integrating aptamer research into a teaching lab, the Aptamer Stream research experience is at its core authentic, with long-term research goals to identify aptamers and develop aptamer applications. Signifying a new endeavor in education, this work describes the use of aptamers in an undergraduate research setting, identifies and characterizes novel anti-calf intestinal alkaline phosphatase (CIAP) aptamers identified in the Aptamer Stream, and highlights some of the student educational benefits from such a research experience.

## 2. Materials and Methods

### 2.1. Integration of Aptamer Research into A Teaching Lab

The integration of aptamer research into an educational setting is at the forefront of all aspects of the Aptamer Stream. In designing the course, the instructional team identified the learning objectives and skills that reflect authentic research practices. These include an emphasis on aptamer research and 21st century learning skill development. For example, in the fall of 2018, students in the Aptamer Stream were assessed on the development of the following skills through the described learning objectives [20]:

1. A fundamental awareness and early experience in scientific research, specifically in the field of aptamer development (oligonucleotide affinity reagent development). This involves an introduction to the terminology, technical concepts, and principles of the research. The learning objectives include:
  - Identify a creative, focused, and manageable research question or topic.
  - Design a methodology for answering a research question, perusing the project, or small-scale “troubleshooting” tests.
  - Demonstrate the understanding of the research implications and its translation to practical applications.
2. Teamwork
  - Brainstorm troubleshooting and/or problem-solving ideas with other students and/or mentors.
  - Make changes to work based on critical analysis of work and on peer review feedback.
3. Communication
  - Develop and practice scientific writing skills.
  - Develop science communication skills, as well as further develop argumentation skills, including the connection between the problem and the solution.
4. Data Analysis Experience
  - Construct a meaningful figure using research data, which includes appropriate controls and statistics, if appropriate.
  - Collect, interpret, evaluate, provide context, and rational conclusions for research data.
5. Resilience
  - Develop and implement mechanisms to overcome, bypass, and/or wade through setbacks.
  - Initiate projects or activities with set deadlines and sometimes incomplete information.

**Figure 1:**
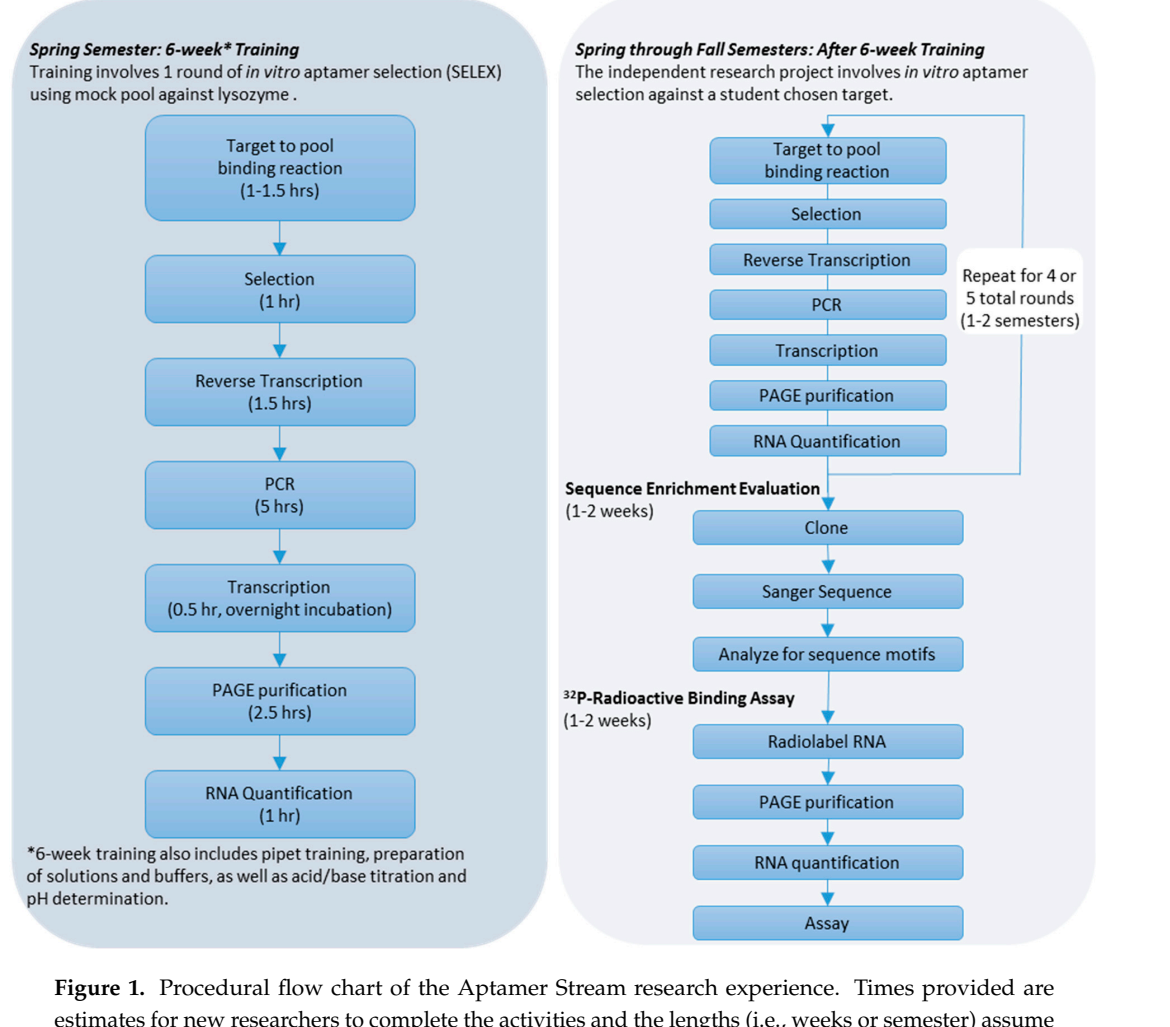
Procedural flow chart of the Aptamer Stream research experience. Times provided are estimates for new researchers to complete the activities and the lengths (i.e., weeks or semester) assume the student researchers work 6–8 hrs/wk with 14 weeks in a semester.

Through the Aptamer Stream two-semester experience, students (approx. 35 students/semester) are trained on the technical aspects of *in vitro* aptamer selections in the first semester. Students work in groups of two (preferably) or three. Midway through the first semester, students, working individually, choose one target from a list of options to begin their independent research project to identify aptamers against that target. The list of options, for example, has included the following targets: glucose oxidase, *Burkholderia pseudomallei* recombinant proteins, dihydrofolate reductase, DNA polymerases, fibroblast growth factors, and horseradish peroxidase. The *in vitro* aptamer selections continues through the summer (optional) and the following fall semester to conclude the formal course sequence (summarized in Figure 1 flow chart). Many students, however, continue their research experience as independent researchers and sometimes in addition to serving as near-peer mentors for the following student cohort. 

The technical piece begins the first semester and involves the iterative *in vitro* RNA aptamer selection process using bead-based (example described below) and/or filter-based methodologies [21] (see Figure 1 for general work flow chart). The training in the aptamer selection involves a 6-week guided laboratory practical through a single round of bead-based *in vitro* aptamer selection. Students use a “mock” RNA pool, prepared through the collection of washes from prior aptamer selections, and select against lysozyme.

Hen egg white lysozyme (catalog no. L6876-5G, Sigma Aldrich, St. Louis, MO, USA) is an ideal target for the training, as it is affordable, available in large quantities, positively charged, therefore electrostatically favorable by the negatively charged RNA, and, perhaps most importantly, known to generate aptamers [22,23]. Prior to the student experience, lysozyme is biotinylated by trained researchers (typically peer mentors) at an 8x molar concentration of EZ-Link Sulfo-NHS-Biotin reagent (catalog no. A39258, Thermo Scientific, Rockford, IL, USA) to lysozyme and purified using a Zeba desalt spin column (catalog no. 89882, Thermo Scientific). 

Briefly, the aptamer selection begins with the immobilization of 200-400 pmol of biotinylated lysozyme on streptavidin paramagnetic beads (Dynabeads M-270 streptavidin, catalog no. 65305 Thermo Fisher Scientific, Waltham, MA, USA) and then exposure to 200-400 pmol of “mock” RNA pool to the target. After a series of washes, the lysozyme-bound RNA is eluted using hot water. The retrieved RNA in the washes and elution is then subjected to ethanol precipitation and then reverse transcribed, generating ssDNA. The ssDNA is used as template in a small-scale PCR reaction to ascertain the necessary cycles for sufficient amplification, as well as suggest the stringency of the selection. That is, washing away too many RNA binders in an overly stringent selection may remove potential aptamers, while washing away too few binders many prolong or even hinder a selection. Once the appropriate number of PCR cycles is determined and selection stringency is appropriate, then students proceed with a large-scale PCR reaction and subsequence ethanol precipitation to concentrate the dsDNA product. This dsDNA is used as template in a transcription reaction. The resulting RNA is then purified via polyacrylamide gel electrophoresis (PAGE) and UV-shadowing. RNA is eluted overnight, ethanol precipitated, and the quantified using the absorbance at 260 nm and the Beer-Lambert equation.

In general, after four to five rounds of RNA aptamer selection, the selected RNA pools are cloned and Sanger sequenced to examine sequence or motif enrichment. ^32^P-radiation binding assays are performed (described in [24]). (For specific information regarding the selection of the anti-CIAP aptamer, please see “Materials and Methods: Anti-CIAP” sections below.)

To meet the educational objectives of the lab, the Aptamer Stream students meet weekly in a lecture-style class, weekly in less formal small group meetings, as well as 6 or 8 hrs/wk in lab in the spring and fall semesters respectively. Weekly lectures in the spring include content-specific lectures related to the *in vitro* aptamer selection methodology (e.g., PCR, gel electrophoresis, oligonucleotide purification and quantification, transcription, etc.), as well as aptamer applications and technologies. In the fall, weekly lectures occasionally include aptamer content (e.g., characterization, applications, etc.); however, most of the time is devoted to student research presentations. The PowerPoint research presentations focus on the student’s independent research project, which includes the theoretical framework for the aptamer application and the progress in their aptamer selection. Some examples of student projects include, “Therapeutic RNA aptamers against Calf Intestinal Alkaline Phosphatase,” “Anti-CIAP Aptamer Utilized in ELISA/ELAA Diagnostic for Multiple Sclerosis,“ and “Aptamer Against Glucose Oxidase for Glucometer Modification to Aid Diabetic Patients.”

In the first semester (spring), students complete assignments assessing their mastery of the content knowledge and lab practices. For examples, students complete “Solution Chemistry,” “Buffer Chemistry,” and “Cloning” assignments, which require students to record and interpret data, reflect and discuss lab activities, as well as answer guiding questions. While conducting the anti-lysozyme aptamer selection lab training, the “Bead-Based RNA Selection” and “PCR and Transcription” assignments instructionally scaffold a larger NIH-style research report. Midway through this semester, when the students move to their independent research projects, students complete two progress reports and a final report, which, specific to their target, resemble the format (and in some cases content) of the reports completed earlier. Along the way, students complete pre-lab quizzes over the provided background materials, maintain a laboratory notebook and organized reagent/sample boxes (i.e., freezer and room temperature boxes). During the fall semester, students generate progress reports and two oral presentations, with an option of substituting one of their PowerPoint talks for a poster presentation.

A key feature of the writing assignments is the opportunity for peer-review and revision. This is important, because technical writing skills for most undergraduate students are still in development. From our experience, reviewing the work of others, as well as the opportunity to revise work based on feedback, noticeably improves the quality of student work. Therefore, most work is peer-reviewed and revised before the final graded submission. 

The Aptamer Stream resides in a 1500 sq ft (approx.) FRI lab space that houses two FRI CURES (i.e., Aptamer Stream and Virtual Drug Screening Stream), as well as the equipment necessary to conduct molecular biology research. The space accommodates 25 to 30 (approx.) people at one time and includes room for instrumentation, bench space for 32 researchers, one fume hood, and one biosafety cabinets adequate for BSL2 work. The laboratory is equipped with one orbital shaker for bacterial growth, an Allegra X15-R centrifuge (Beckman Coulter, Indianapolis, IN, USA) for oligonucleotide pool preparation and precipitations, as well as bacterial and protein preparation, two micro-spectrophotometers (nanodrop spectrophotometer) for sample quantification, a Synergy HT plate reader (96-well, BioTek Instruments, Winooski, VT, USA), a Savant ISS110 SpeedVac Concentrator (Thermo Fisher Scientific, USA), 10 thermocyclers (various manufacturers), four bench-top centrifuges, an incubator, 11 microcentrifuges (various manufacturers), two analytical balances, multiple pH meters, multiple heat plates, seven benchtop vortexers, four benchtop water baths, two Max Q 7000 water baths, (Barnstead International, Dubuque, IA, USA) multiple gel electrophoresis apparatuses an ultrafreezer, a nanopure water dispenser (Barnstead International) and all the refrigerators, and materials necessary for molecular biology research. Course protocols, handouts, and supportive educational materials are available upon request to the corresponding author.

### 2.2. Anti-CIAP Aptamer Research: in Vitro Aptamer Selection (RNA Aptamers)

The *in vitro* anti-CIAP aptamer selection was conducted by an Aptamer Stream student Vincent Huynh (co-author, since graduated). The generalized and annotated selection protocol provided to the class was followed. Huynh chose the aptamer selection conditions (i.e., buffer, incubation temperature, time, etc.), as well as adapted his aptamer selection to maintain an appropriate stringency, thus washing away non-binding RNAs and enriching CIAP-binding RNA. The details of Huynh’s anti-CIAP aptamer selection are described below.

In general, biotinylated-CIAP (69 kDa, catalog no. 29339 Pierce Thermo Fisher Scientific) and pool were employed in a toggle selection, using streptavidin bead-based selection and a filter-based selection methodology [21] to enrich anti-CIAP aptamers. The N50 RNA pool (97 nt) was used in the *in vitro* anti-CIAP aptamer selection, 5′-GGGUUUACCUAGGUGUAGA UGCU-N50-AAGUGACGUCUGAACUGCUUCGAA-3′. Refer to Table S1: Anti-CIAP Aptamer Sequences for a text file of all oligonucleotides used in this research.

For example, in round 1, 50 μL of streptavidin beads (Fisher Dynabeads M-270 Streptavidin, catalog no. 65305) were washed with water, then incubated for 30 min at room temperature with 400 pmol of biotin-CIAP, and then washed with PBS. After heat denaturing and cooling to room temperature, the 400 pmol of natively folded N50 RNA pool was incubated with the CIAP-beads (round 1) at room temperature for 25 minutes (rounds 1-6) or 15 minutes (rounds 7-9). After the incubation, several washes with selection buffer (20 mM HEPES, pH 7.5, 150 mM NaCl, 5 mM MgCl_2_) were performed to remove the weakly bound RNA species. For round 1, the total wash volume was 2.4 mL. In an effort to minimize the non-specific RNA binders and enrich CIAP-specific RNA binders, the selection stringency was increased each round by increasing wash volumes, performing negative selections, as well as increasing the selected RNA to CIAP ratio (i.e., decreasing CIAP from 400 pmol to 50 pmol). After the last wash, a negative selection was performed by removing the CIAP-beads and RNA into a new tube, thus removing RNA plastic tube-binders. RNA bound to the CIAP was eluted using 80 °C nanopure water. Negative bead selections were performed every round (except 1 and 5) prior to incubation with the CIAP-beads by exposing the RNA pool to naked beads (i.e., without CIAP), thus removing bead-binding RNA. The collected washes and eluted RNA were ethanol precipitated. See Appendix A, Table 1 for selection conditions (i.e., wash volumes, negative selections, etc.) for each round.

To amplify and analyze the RNA pool collected in washes and eluted for each round, the DNA analog was prepared and amplified. The selected N50 RNA pool was subjected to a reverse transcription reaction with the reverse primer “24.N50.R” primer 5′-TTCGAAGCAGTTCAGACGTC ACTT-3′

The subsequent cDNA was then amplified in various PCR reactions, which included a forward primer that reappended the T7-promotor (T7-promoter noted in bold): “42.N50.F” primer 5′-GA**TAA TACGACTCACTATA**GGGTTTACCTAGGTGTAGATGCT-3′

In general, a cycle course PCR [24] was used to determine the effective ratio of non-binding RNA to CIAP-bound RNA (thus evaluating the stringency of the selection), as well as determine the necessary PCR cycles for sufficient amplification. A subsequent large-scale PCR reaction was performed and then concentrated via ethanol precipitation. 

Recapitulating the selected RNA, a transcription reaction was performed for 2 hours at 42 °C or overnight at 37 °C using a T7 RNA polymerase. RNA was PAGE purified using UV shadowing. Finally, the selected RNA was eluted from the PAGE gel chunks overnight in 10 mM Tris-HCl, pH 8.0 and 1 mM EDTA (TE) buffer or by the crush soak method, subsequently ethanol precipitated, resuspended in selection buffer, and spectrometrically quantitated in preparation for the next iterative round of aptamer selection.

### 2.3. Anti-CIAP Aptamer Research: Sanger Sequencing and Aptamer Binding Assay of Selected Pool

Using a TA Cloning kit (catalog no. K2020-20, Thermo Fisher Scientific Invitrogen,), PCR product from selected rounds was cloned into the pCR 2.1 vector and Sanger sequenced using M13 primers. Sequence enrichment was determined by visually inspecting sequence data for recurring clones and motifs.

To ascertain the RNA to CIAP binding enrichment over multiple rounds of aptamer selection, a ^32^P radiation binding assay was performed similarly to that described previously [24]. Recurring RNA clones in the selected pool (i.e., potential aptamers), as well as the minimized variant, were tested as in such a binding assay.

### 2.4. Creating a Culture of Safety

Working with students new to research, the Aptamer Stream approaches safety in a holistic, on-going fashion. The first step to this approach involves building familiarity with the lab hazards and safe use of these materials. For example, unpolymerized acrylamide (a neurotoxin) is used to generate polyacrylamide gels to purify RNA transcripts. Ethidium bromide (mutagenic and potentially carcinogenic) is used to visualize DNA on agarose gels. Lastly, acids and bases are occasionally used to adjust the pH of buffers. Ethidium bromide, acrylamide, acids and bases are localized to specific areas in lab, dedicated waste bins are used for disposal, and full lab attire is required when working with the material (i.e., lab coat, goggles, and gloves). UV light (dangerous to eyes, mutagenic, and carcinogenic) is regularly used to visualize gels. Training and regular assistance is provided when working with UV lights, which, too, require the use of full lab attire.

Further developing a continued approach to safety, every lecture begins with a “Safety Minute” with a reflection, discussion, or short activity devoted to safety or ethics in the laboratory. Below are some examples of “Safety Minute” discussion prompts:

- In an effort to preserve a culture of safety in the lab, let’s talk about how to protect ourselves in lab. Where do we find personal protective equipment (PPE) in lab and when do we need to wear it?
- Here are a few safety reminders when performing polyacrylamide gel electrophoresis (PAGE): Unpolymerized acrylamide is a neurotoxin and must be kept in the PAGE hood area. Polymerized acrylamide (i.e., solid-gel acrylamide) may be safely disposed of in the regular trash can. If you break a PAGE gel glass, please report it to a mentor and dispose of the glass in the glass waste box. How do we remember these things? Can you create a picture/cartoon, song, or poem to remind us of these safe PAGE practices?
- Sometimes it’s the smallest things. “Hot glass looks like cold glass!” –Alex G. When microwaving agarose solutions, please loosen the lid. Remove clutter from the area to clear a spot to eventually place the hot bottle. And - use a hot pad to remove the bottle from the microwave. Can you think of other "small things" in lab that could be potential safety hazards and what actions can be done to mitigate the hazard potential? Please share one or more idea with your neighbor. You’ll have an opportunity to share your idea with the class.

Before lab work begins, all students must complete University-designed safety courses: Hazardous Communication, Laboratory Safety, and Hazardous Waste Management. Subsequent courses are required later in the experience, such as the Fire Extinguisher Training course and the Basic Radiological Health Training (if performing a radiation binding assay). Additionally, every semester, all students complete a “Site-Specific Training” course, which (re-)identifies the hazards in lab, discusses safe practices, as well as the emergency response protocols. 

### 2.5. Educational Assessment Methods

To track and monitor the impact of this authentic research experience on students, both formative and summative assessments are conducted each semester and annually at both the research stream level and at the FRI programmatic level. The formative assessments provide instructors with student feedback to gauge what is working well and what needs improvement. The data gleaned from feedback forms conducted each semester inform any mid-course corrections and provide instructors with actionable information to enhance the experience for students. The summative assessments examine how the experience in the aptamer research stream impacted students’ psycho-social attitudes towards science and science research. Pre/post surveys are administered each semester to track student growth over time. Specifically, the survey draws on previous, empirically validated measures designed to assess: Science Self-Efficacy [25], Identity and Belonging in Science [25,26], Grit/Resilience [27,28], and Intention to Persist [29] and Preparedness [27]. 

In addition to assessing psychosocial attitudes, the pre/post survey also captures students’ self-reported gains in research skills from pre to post. Specifically, the following 21^st^ Century Learning Skills [30] were assessed:

- *Effective Communication*: the ability to produce written and oral reports, and make persuasive, evidence-based arguments using appropriate scientific sources and effective figures and graphics.
- *Information Literacy*: the ability to locate appropriate information, evaluate sources critically, read and interpret primary scientific literature, and synthesize information.
- *Computational/Technological Literacy*: the ability to organize and interpret data and apply computational skills to solve problems.
- *Self-directed Learning*: the ability to execute an independent and original project culminating in a product, such as a written document, oral presentation, or physical object; the ability to innovate, create, or conduct original research projects.
- *Teamwork*: the ability to resolve conflicts, plan, and coordinate group efforts.

In the fall of 2017, a pre/post in-class student survey was deployed to approximately 24 students participating in the Aptamer Stream. The survey included items reflecting the constructs described above (e.g., Science Self-efficacy: “I have a lot of confidence when it comes to doing STEM research.”). Students were asked to rate each item on a 5-point Likert scale (1, Strongly Disagree to 5, Strongly Agree). The data gleaned from the surveys were used to both track students’ growth over time and to assess the extent to which the learning objectives of the course were met (e.g., Skill: Teamwork). 

## 3. Results

### 3.1. Anti-Calf Intestinal Alkaline Phosphatase (CIAP) Aptamer Research Results

#### 3.1.1. Anti-CIAP Aptamer Selection and Sequence Enrichment Results

Nine rounds of *in vitro* aptamer selection using an N50 RNA pool were performed to identify RNA aptamers against calf-intestinal alkaline phosphatase (CIAP). Early radiation binding assays revealed CIAP-binding enrichment in selected RNA from rounds 4 and 6 over round 1 (see Appendix B, Figure 1). Subsequently, three additional rounds, nine rounds in all, of *in vitro* aptamer selections were performed with a decreased RNA:CIAP incubation time and increased stringency (see Appendix A, Table 1), further challenging the CIAP-binders with increased washes and increased RNA pool: CIAP ratio.

The selected RNA pool from round 9 was cloned, Sanger sequenced, and the sequencing data was visually inspected for sequence enrichment through the presence of recurring clones and sequence motifs. Of the 36 clones examined, two of the RNA clones were present twice and with a 13 nt motif (GAACUCAACAUAA) present in 19 of the examined sequences. Of these 19 sequences, 15 contained a G at the end of the motif and 4 contained an A at the end, thus the motif was extended to a 14 nt motif with the representation of a punctuating purine (GAACUCAACAUAA**R**). When folded, 18 of the 19 clones containing the recurring motif present it in the loop structure (see Table 1). 

#### 3.1.2. Anti-CIAP Aptamer Minimized Variant Design, Assay Results, and Dissociation Constants

**Figure 2:**
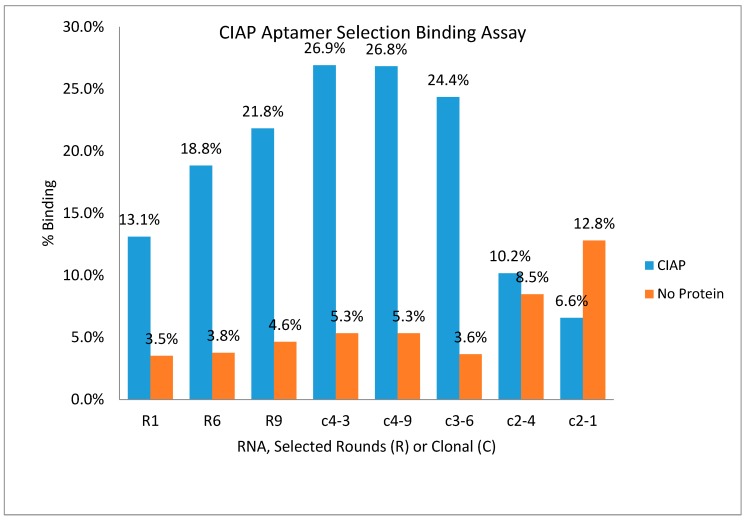
Anti-CIAP aptamer selection radioactive binding assay results (representative experiment without replicates). RNA clones c4-3, c4-9, and c3-6 contained the 14-mer motif and had the greatest binding affinity to CIAP. RNA clones c2-4 and c2-1 did not contain the 14-mer motif and, as observed in the graph, had the lowest binding to CIAP and the highest binding to beads (“no protein”).

**Figure 3:**
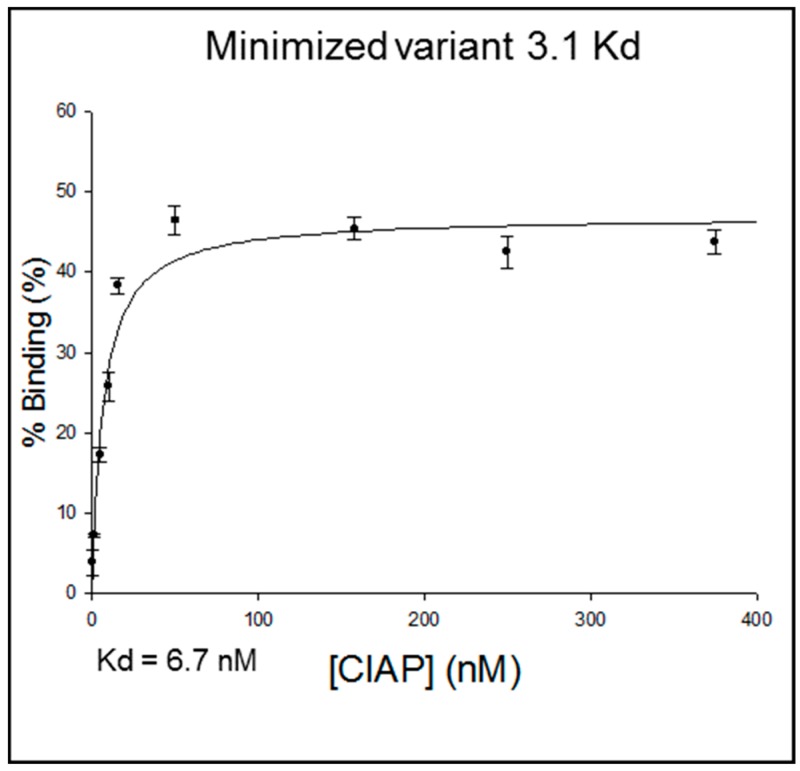
Dissociation constant (Kd) of minimized anti-CIAP RNA aptamer variant 3.1 and CIAP.

Testing selected RNA pools (i.e., rounds 1, 6, and 9) and recurring clones in a ^32^P radioactive binding assay revealed increased CIAP-binding, relative to the “no protein’ beads, over multiple rounds (see Figure 3). RNA clones c4-3, c4-9, and c3-6, which contained the 14-mer VDH motif, demonstrated significant CIAP-binding over the “no protein” beads, binding more than the round 9 selected RNA pool. However, clones without the VDH motif (e.g., c2-4 and c2-1, see Table 2) bound did not bind CIAP above background/”no protein” beads (see Figure 2) and were thus eliminated as potential CIAP aptamers (Table 2).

In an effort to rationally design a minimized binding variant, clone 4-3 was chosen from the three clones tested (i.e., c4-3, c4-9, and c3-6, see Table 2) because initial binding assays indicated the greatest percentage of CIAP-binding from this clone (Figure 3). Using the mFold structure [31], the minimized variant was designed to preserve the VDH motif and the neighboring structural features (i.e., the primary loop and stem), while minimizing the distal structural elements. 

Given this design approach, the minimized variant 3.1 contained approximately more than half of the 5′ pool static region (aka forward primer region), most of the random region, and none of the 3′ static region (aka reverse primer region). Furthermore, to enhance transcription using a T7-promoter, a GGG was added at the beginning of the construct with a complementary CCC at the 3′ end, thus contributing to the stem structure (see Table 2), and forming the minimized variant 3.1.

Using ^32^P-RNA binding assay, the dissociation constant of the minimized variant 3.1, as well as clones c4-3, c4-9, and c3-6, was determined to be 6.7 nM (see Figure 3), 5 nM, 9.4 nM, and 10.8 nM, respectively (Appendix C). 

#### 3.1.3. Anti-CIAP Aptamer Specificity and Activity

Assaying for binding specificity, the aptamer binding affinity against many CIAP-related proteins (such as human intestinal AP, human tissue-nonspecific AP, and bacterial AP) was examined and no significant binding was observed in radioactive binding assays up to 250 nM protein concentrations (see Appendix D). Early kinetic assays examining aptamer inhibitory effects on the reaction of CIAP and its substrate *p*-nitrophenyl phosphate indicate little if any loss in alkaline phosphatase activity (data not shown). A follow-up kinetic study is suggested to conclude these findings.

### 3.2. Results of Student Assessment Outcomes

Using survey data collected in Fall 2017, paired samples t-tests were utilized to assess Aptamer Stream student gains from pre to post across attitudes (e.g., self-efficacy) and 21st Century Learning Skills (e.g., effective communication). Fourteen out 24 aptamer students completed the pre/post student survey in Fall 2017 and consented to include their responses for research purposes. Given the small sample size, caution should be employed when interpreting the data as the findings displayed are considered preliminary only. Increasing the sample size and collecting data across multiple semesters is needed to validate and replicate these findings. 

**Figure 4:**
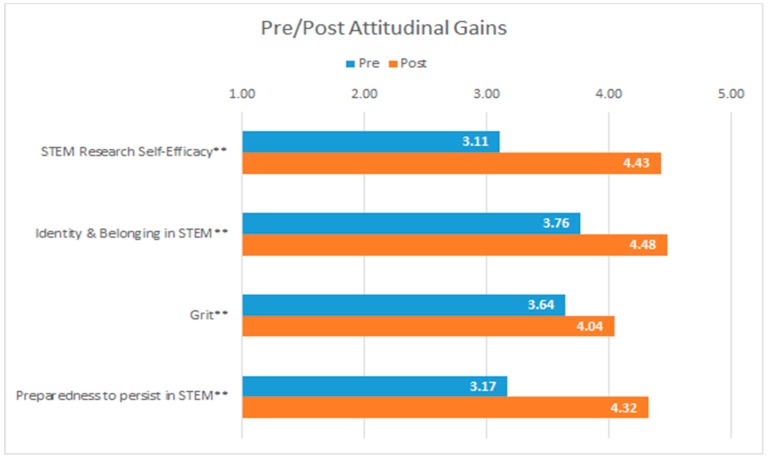
Pre/post attitudinal gains across 14 students participating in the Aptamer Stream in Fall 2017. Scale: 1, Strongly Disagree to 5, Strongly Agree. ** *p* < 0. 01, * *p* < 0.05. The data displayed above capture construct averages.

**Figure 5:**
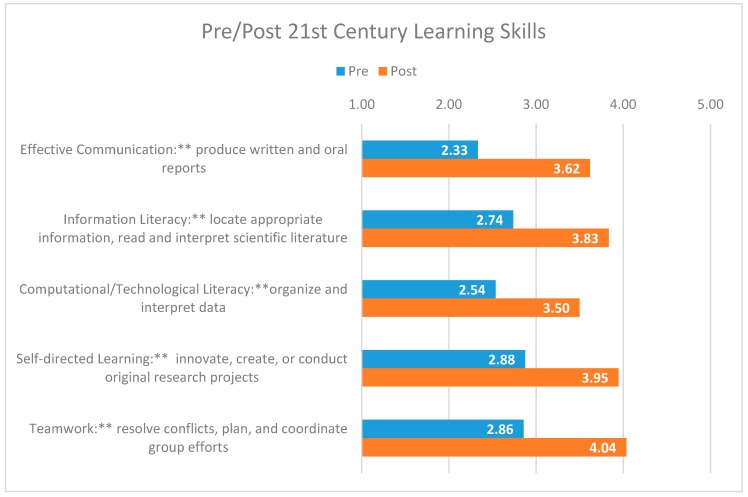
Pre/post research skill gains across 14 students participating in the Aptamer Stream in Fall 2017. Scale: 1, Poor to 5, Excellent. ** *p* < 0.01, * *p* < 0.05. The data displayed above capture construct averages.

The findings, displayed in Figure 4 and Figure 5, suggest that students express gains in both their attitudes and research skills from pre to post. Among students’ attitudinal gains (Figure 4), the data suggest that students express statistically significant improvements in their research self-efficacy (e.g., “I have the skills to conduct my own STEM research project.“), identity and belongingness (e.g., “I feel like I belong in STEM.”, grit (e.g., “When I am working on a research problem that I can’t immediately understand, I work harder to find a solution.”), and preparedness to persist in STEM (e.g., “I feel prepared to do advanced coursework in STEM.”). The largest gains were in research self-efficacy: before participating in the Aptamer Stream, students rated themselves a mean of 3.11 (Std. dev: 0.83) on a 5-point Likert scale; after participating in the Aptamer Stream the mean increased to 4.43 (Std. dev: 0.53). 

Among their 21st Century Learning Skills (Figure 5), the data further suggest that students show statistically significant increases in their self-reported ability to communicate effectively (e.g., write scientific reports), locate and interpret information (e.g., read and interpret information in scientific literature), interpret and analyze data, engage in self-directed learning (e.g., conduct research independently), and work effectively on a team (e.g., communicate effectively with lab and research partners). Together, this provides preliminary evidence to suggest that the aptamer research experience provides students with the skills, confidence and know-how to conduct research within a lab setting and undertake their own independent research projects. 

For formative purposes, the survey included a few open-ended questions and a satisfaction rating scale to further offer insights into students’ general perceptions of the research experience. In terms of general satisfaction, 77% of students indicated that they would “very likely (5)” recommend participating in the aptamer experience to other students (scale: 1, very unlikely to 5, very likely). When asked to elaborate on their satisfaction rating, students indicated that they most valued and benefited from the hands-on learning experience of designing and executing their own research project. For example, one student said, 

“[This experience] allows us to truly delve into a research project of our own design, and forces us to think critically in a way no other lab can provide.” 

Another student highlighted the lab skills that they learned in the stream and the uniqueness of this experience as a first-year STEM student: 

“I think that it is very valuable to learn lab techniques and skills such as gel electrophoresis or PCR as an undergraduate because we are exposed to research before we enter graduate school or upper division courses. Not many universities allow freshman/sophomores to be completing research in real labs so I think this is a very unique opportunity UT offers to the students.”

Overall, the preliminary survey data suggest that the Aptamer Stream provides students with an authentic hands-on learning environment whereby their attitudes and research skills are being cultivated and enhanced over time. While pre/post survey data provides some data to suggest that students express gains across key areas of development, it may not necessarily point to the efficacy of the aptamer stream in particular. That is, without a comparison group, the gains in attitudes and skills could be attributed to a maturation effect— a natural improvement in cognitive abilities that takes place during the college years. On-going education research comparing aptamer students to a matched comparison population are currently underway, and will further test the impact of this experience controlling for a possible maturation effect.

## 4. Discussion

Integrating teaching and learning objectives into a research lab, the resulting Aptamer Stream lab yields positive outcomes in aptamer generation and student attitudes and research skills. This manuscript serves as a testament to positive research contributions, as well as the positive student outcomes. 

Point in case, one of the early positive research outcomes was the identification and characterization of novel anti-CIAP RNA aptamers with nM affinities for CIAP. (Specifically, the anti-CIAP RNA aptamers 4-3, 4-9, 3-6, and minimized 3-1 variant have the following Kds, respectively: 5 nM, 9.4 nM, 10.8 nM, 6.7 nM.) After the one year CURE in the Aptamer Stream, undergraduate Vincent Huynh (co-author) identified anti-CIAP aptamers after nine rounds of *in vitro* aptamer selection. Through Huynh’s remaining sophomore to senior year at The University, the anti-CIAP aptamers were studied, characterized, and used in the development of assays. While the assay development continues, Huynh’s research has provided many notable contributions, including the establishment of radiation binding assay positive controls (i.e., the anti-CIAP aptamer and CIAP pair), development of teaching lab best practices and the sequence of aptamer analysis and characterization, as well the connection and development of a network of resources and expertise (e.g., Kd assay and analysis, activity assays, etc.). Furthermore, the anti-CIAP aptamers have the potential to be used in ELISA or ELONA assays, which, for example, could non-covalently connect the CIAP reporter molecule to a detection aptamer/antibody. Such an application could lead to forgoing the potentially costly chemical conjugation of detection aptamers or antibodies to instead use a simple, non-covalent reporter system.

None of these research contributions, however, would have been possible without the careful consideration of the environment, which included not only research priorities, but educational commitments. Briefly described here, this includes the consideration of the student, in general the students’ background knowledge, interest, timeframe, as well as the minimal budget, equipment, and material needs for a research course (Table 3). Joining the Aptamer Stream in the second semester of their freshman year, most student researchers had little to no background in molecular biology or biochemistry (such as nucleic acid functionality, molecular biology techniques-PCR, gel electrophoresis, nucleic acid quantification, etc., assay development, etc.). However, student interest in the aptamer-field, especially in the area of generating medical diagnostics and therapeutics, in general is fairly high. This is most evident in that more students request this FRI stream/course than there are seats available and that the retention of students from the spring to the fall semester is high (>75% over multiple years). Additionally, in an effort to accommodate the undergraduate students’ schedules, which are typically full of courses, student organizations (e.g., pre-medical/health organizations, mentoring organizations, honor societies, and organizations fundraising for a cause, etc.) and volunteering experiences, the lab operates approximately 9a to 6p M-F with undergraduate near-peer mentor support. This open-door policy provides the flexibility for students to conduct lab work within their own schedules.

With respect to minimizing equipment and materials expenses, this is an area fraught with creativity and innovation. Briefly, and for example, enzymes (such as *Taq* DNA polymerase and T7 RNA polymerase) are acquired through in-house preparations, collaborators, or even in the form of raw “cellular reagents” (i.e., lyophilized cell lysate containing overexpressed enzymes) [32] (see Table 3). While the cost of these enzymes are minimized, batch-to-batch activity variations introduces error and necessitates quality control measures, as well as an intentional focus on teaching such topics as “experimental controls” and “experimental design.” To minimize equipment costs, cycle course PCR is utilized (in lieu of real-time PCR) to estimate the number of PCR cycles need to sufficiently amply selected pool. As a last example of cost-saving measures, new and more affordable materials (such as streptavidin-conjugated beads) are regularly tested and integrated into the lab. 

Additional considerations in the development of research projects include the likelihood of project success. FRI streams are led by non-tenure track faculty members (Research Educators) with milder requirements to publish than tenure-track faculty, thus the streams have an opportunity to take on risky projects with a small likelihood of success. Furthermore, aptamer selections using canonical nucleotides have a small likelihood of success. SomaLogic reported < 30% of their aptamer selections against human proteins were successful when using RNA, DNA, or 2’-fluoro-pyrimidine RNA [33,34]. Further compounding the challenges of the research experience, the Aptamer Stream marks many students’ first encounter with research. However, there may be some advantage to providing challenging, yet attainable research goals, which, when met or nearly met, may promote positive attitudes towards science. In so much, some of the Aptamer Stream research projects were designed with this in mind, such as projects seeking the identification of *novel* aptamers against targets with known aptamers, thus validated aptamer targets (e.g., anti-lysozyme [22,23]), anti-CIAP [35], etc. aptamer projects). For example, CIAP has known aptamers [35], which predate Huynh’s aptamers. 

While the UT Aptamer Stream (est. 2006) was seemingly the first course-based undergraduate experience in aptamer research and served as an early model for such an experience, another aptamer CURE was recently established. The University of Maryland First-Year Innovation and Research Experience (FIRE) Engineering Biosensors Research Stream was launched in 2018. This undergraduate teaching and research lab specializes in “selecting and characterizing aptamers” and “designing and testing aptamer-based biosensors” [11]. Building a community of aptamer education, students in the FRI Aptamer Stream and the FIRE Engineering Biosensors Research Stream share a common blog, as well as peer review materials, in an effort to build collaboration and science communication skills. With the emergence of the FIRE Engineering Biosensors Research Stream and ideally new aptamer CURES, there is a potential for collaboration, sharing of resources and methodologies, and, most importantly, integrating students into the research experience.

In reflection on the Aptamer Stream to serve both research and educational objectives, a question about the “defining” features of the course are raised: Is there something about aptamer research that is intrinsically well-matched for an educational environment? Auchincloss et al. [36] proposed five essential features of CUREs: scientific practices, iteration, discovery, relevance/broader impact, and collaboration. The iterative process of *in vitro* selection methodology, sieving of libraries in discovery-based research, broadly applicable aptamers (e.g., therapeutics, diagnostics, and sensor applications), and now the new multi-institutional Aptamers in Education community, which are all central to the work, speak to the natural fit of aptamers into the education realm of course-based undergraduate research experiences. This type of research experience is increasingly important for students to learn (from virtually their first days on campus) that they can manipulate biology themselves, and serves as a powerful springboard for a variety of professional experiences, from medical school to graduate school to direct entry into the biotechnology industry. Perhaps this new approach to education and aptamer research will open the doors to similar types of research experiences, benefitting both the aptamer community and undergraduate education as a whole. 

## 5. Conclusions

In summary, the Aptamer Stream research course is a unique junction between aptamer research and education. Positive outcomes in both research and education have been realized and the evidence for such provided. Here, we bring forth novel anti-calf intestinal alkaline phosphatase aptamers, the presumably first-of-its-kind course-based undergraduate research experience in aptamers, and many innovative details describing the merger of the research and educational aims of the experience. In conclusion, we offer this synergistic approach to aptamer research and education as a fruitful model for future undergraduate science education. 

## Figures and Tables

**Table 1:** RNA clones without the VDH 14-mer motif. Binding assay data later eliminated these clones as potential anti-CIAP aptamers as they non-discriminately (or even preferentially in the case of c2-1) bound naked beads (see Figure 2).

Clone	Sequence
c2-4	GGGUUUACCUAGGUGUAGAUGCUUCUUCACUCCUUAUGAACACGUAGCGCUCAAUCAUCUCUAAUUAAUUCUCAAGUGACGUCUGAACUGCUUCGAA
c2-1	GGGUUUACCUAGGUGUAGAUGCUGAUAGAUUUCCCCUGACUUAGGAGCUCAUUAGAUUUUUAUUGUGUGGGGCAAGUGACGUCUGAACUGCUUCGAA

**Table 2:** Recurring clones and sequence motifs identified in the anti-CIAP *in vitro* aptamer selection (round 9). The bold sequences identify the former “random region” of the original N50 RNA pool and the non-bold/regular-type sequences identify the static regions designed for primer annealing. Note the presence of an R (i.e., A or G) in the VDH 2.14 motif and the presence of a W (i.e., A or T) in aptamer clone 4-9. Refer to Table S1: Anti-CIAP Aptamer Sequences for a text file version of this table.

Motif or Clone	% Occurrence (n = 36 clones)	Kd	Fold and Notes	
Motif VDH 2.14, 14 nt: GAACUCAACAUAAR	53%	Not tested	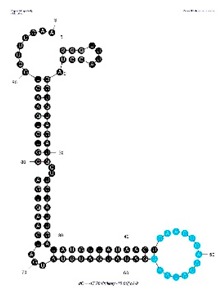	VDH 2.14 motif appears in the loop structure (as seen in c2-2) approx. 18 out of 19 clones. Note the presence of an R (A or G).
Aptamer Clone 4-3, 97 nt: 5′GGGUUUACCUAGGUGUAGAUGCUGUAUAUAGCGAACUCAACAUAAGGUAUAAUUACAAUUUCUAUACUUCUUCAAGUGACGUCUGAACUGCUUCGAA3′	6%	5 nM	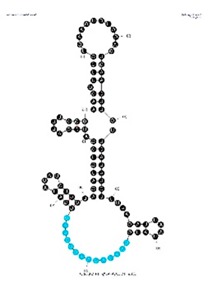	VDH 2.14 motif appears in the loop structure (as seen in c4-3) approx. 2 out of 2 times.
Aptamer Clone 4-9, 97 nt: 5′GGGUUUACCUAGGUGUAGAUGCUUCWAUUGAUAUGUUAUAACUGAACUCAACAUAAGGAUAUGAUGUAUGAUCAAGUGACGUCUGAACUGCUUCGAA3′	6%	9.4 nM	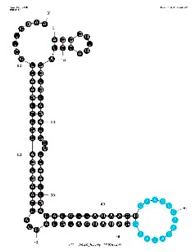	VDH 2.14 motif appears in the loop structure (as seen in c4-9) approx. 2 out of 2 times. Note the presence of a W (A or T).
Aptamer Clone 3-6, 97 nt: 5′GGGUUUACCUAGGUGUAGAUGCUCUGCCCUUCAGAUUUAUCGAUGACCGUUGAACUCAACAUAAGACCUUCCAAAGUGACGUCUGAACUGCUUCGAA3′	3%	10.8 nM	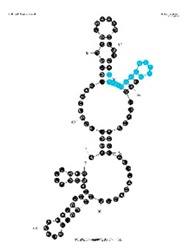	VDH 2.14 motif is present in the loop and stem structure.
Minimized Aptamer Variant 3.1, 55 nt: 5′GGGUAGAUGCUGUAUAUAGCGAACUCAACAUAAGGUAUAAUUACAAUUUCUACCC3′	N/A	6.7 nM	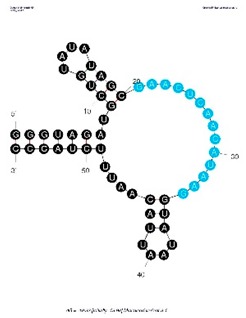	3.1 is a minimized variant of aptamer clone 4-3. The triplet GGG was added to 5′ end to improve transcription efficiency and, thus CCC added to 3′ end to participate in the stem structure, contributing to a lower dG.

**Table 3:** How the Aptamer Stream adapted the research to a CURE/educational environment and considerations for replication.

Research-Education Features of Consideration	Aptamer Stream	Replication of Same or Similar CURE
Student background knowledge	Freshman students, with limited or no experience in aptamer selection laboratory skills or contextual background, are taught both simultaneously in a 1-hr/wk lecture and 6- to 8-hr/wk laboratory work, with additional support mechanisms (e.g., small group meetings, office hours, etc.).	Guided and independent laboratory research opportunities complemented with lecture/contextual background lessons and discussions are suggested.
Student interest	The biomedical potential of aptamers (i.e., diagnostic and therapeutic applications) are well-received by the College of Natural Sciences undergraduates, many/most with plans for a career in a health field.	Matching a research experience/CURE with the student interest and/or career prospects is suggested. Surveying and/or consideration of student career interest is encouraged.
Student time	Students commit at least 6 hrs/wk in the spring semester and 8 hrs/wk in the fall semester to their aptamer research (~14 wks/semester). Students may schedule their own lab hours M-F 9a-6p.	Consideration of the lab hour flexibility, while providing safe opportunities for training, guidance, and oversight (such as through undergraduate peer mentors) is suggested.
Reagents and enzyme	To minimize the cost of reagents and enzymes, reagents are often purchased in bulk or even prepared in lab, if possible (e.g., PCR buffer, plasmid purification reagents, etc.). Enzymes have been provided by other teaching labs, collaborating labs, etc. Additionally, some raw, unpurified enzyme preps have been used, as well [32].	Consider how enzymes and reagents will be affordably purchased. Undergraduate and new researchers may consume, at times, more reagents than experienced researchers, as the learning curve (such as pipetting errors, solution preparation mistake, etc.) are common.
Equipment	Equipment common to most molecular biology labs, with the exception of the speedvac and the plate reader (optional) is sufficient for Aptamer Stream research (see Materials and Methods section). While the speedvac and plate reader are not required, the plate reader is helpful for assay development.	Use caution when obtaining old, well-used equipment, or even flimsy equipment, as novice researchers in their attempts to learn how to use the equipment, may inadvertently damage or induce wear on the equipment. Robust equipment and a maintenance and repair budget is suggested, when possible (such as for micropipette repair/calibration).

**Figure A1:**
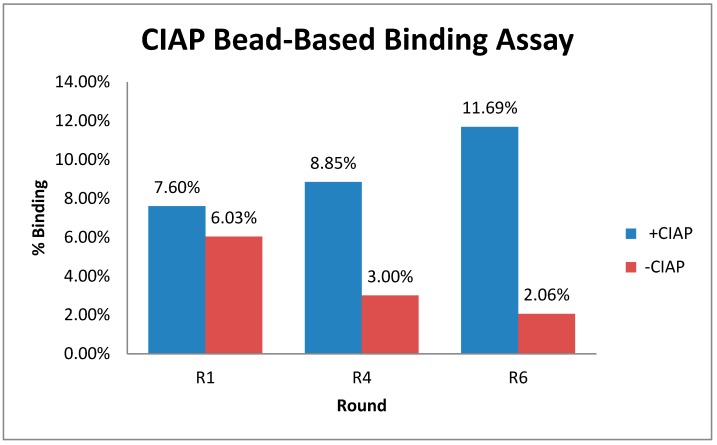
Early anti-CIAP ^32^P-labeled RNA binding assay results for round 1 (R1), round 4 (R4) and round 6 (R6) selected pools against biotinylated-CIAP on streptavidin beads. These early results indicate an increase in selected RNA bound to CIAP over the rounds of selection and a decrease in the binding of the RNA pool bound to naked beads.

**Figure A2:**
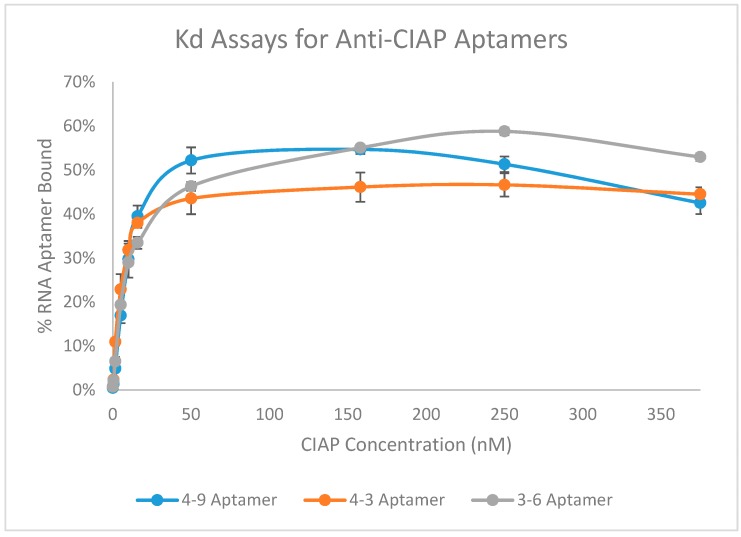
Dissociation constants (Kd) of anti-CIAP aptamers. Kd assays were performed in triplicate using radioactive ^32^P-labeled RNA aptamers (5 nM). From these results, the anti-CIAP aptamers 4-3, 4-9, and 3-6 anti-CIAP aptamers were found to have the following Kds, respectively: 5 nM, 9.4 nM, and 10.8 nM.

**Figure A3:**
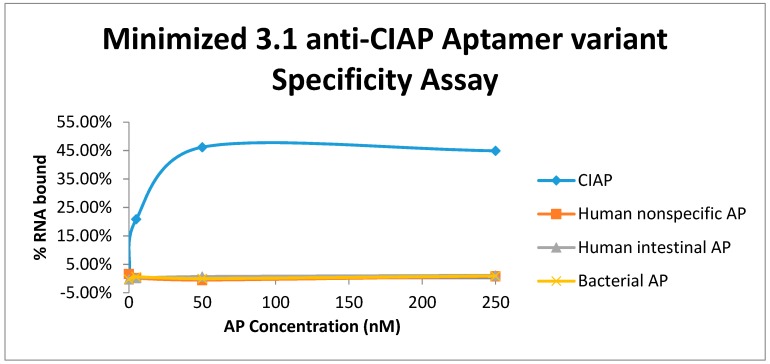
The target specificity assay involved the radiolabeled minimized 3.1 anti-CIAP aptamer variant and binding assays against a variety of alkaline phosphatases, including calf intestinal alkaline phosphatase (i.e., positive control), human tissue nonspecific alkaline phosphatase (recombinant), human intestinal alkaline phosphatase (recombinant), and bacterial alkaline phosphatase (recombinant). In this single point assay (i.e., no replicates), no significant aptamer binding was observed to the non-CIAP targets.

## Supplementary Materials

The following are available online at https://www.mdpi.com/1424-8220/19/15/3270/s1, Table S1: Anti-CIAP RNA Aptamer Sequences, text file. The bold sequences identify the former “random region” of the original N50 RNA pool and the non-bold/regular-type sequences identify the static regions designed for primer annealing. Note the presence of an R (i.e., A or G) in the VDH 2.14 motif and the presence of a W (i.e., A or T) in aptamer clone 4-9.

## Appendix A—CIAP Aptamer *in vitro* Selection Conditions

**Table A1:** CIAP aptamer *in vitro* selection conditions. * indicates a negative selection was performed by removing RNA species that bound naked beads (i.e., beads without CIAP).

Round	RNA:CIAP (pmol:pmol)	Volume of Beads Used (μL)	# of PCR Cycles Necessary to Amplify Selected Pool	Total Wash Volume (mL)	Amount of Recovered Pool (pmol)
Round 1	400:400	50.0	10	2.4	2093
Round 2^*^	400:400	50.0	14	2.4	388
Round 3^*^	300:300	44.1	11	2.4	301
Round 4^*^	120:100	14.7	15	3.2	969
Round 5	200:100	14.7	9	3.2	906
Round 6^*^	200:100	14.7	9	4.0	298
Round 7^*^	200:50	7.35	9	6.0	308
Round 8^*^	200:50	7.35	13	13.9	1241
Round 9^*^	800:50	7.35	13	47.2	735
