# Supplementary material for: Aptamers in Education: Undergraduates Make Aptamers and Acquire 21st Century Skills Along the Way
Source: Sensors (Basel). 2019 Jul 25;19(15):3270. doi: 10.3390/s19153270 (PMC6696043; doi:10.3390/s19153270)
Supplement: Supplementary file 1 [file sensors-19-03270-s001.pdf]

Aptamers in Education: Undergraduates Make Aptamers and Acquire 21st Century Skills Along the Way

Gwendolyn M. Stovall [1,2,*], Vincent Huynh [1], Shelly Engelman [3] and Andrew D. Ellington [4]

1. Texas Institute for Discovery Education in Science, Freshman Research Initiative, University of Texas at Austin, Austin, TX 78712, USA
2. Texas Institute for Discovery Education in Science, High School Research Initiative, University of Texas at Austin, Austin, TX 78712, USA
3. Texas Institute for Discovery Education in Science; University of Texas at Austin, Austin, TX 78712, USA
4. Institute for Cellular and Molecular Biology, University of Texas at Austin, Austin, TX 78712, USA

* Correspondence: gwenstovall@utexas.edu; Tel.: +01-512- 814-6176

Table S1: Anti-CIAP RNA Aptamer Sequences, text file. The bold sequences identify the former "random region" of the original N50 RNA pool and the non-bold/regular-type sequences identify the static regions designed for primer annealing. Note the presence of an R (i.e. A or G) in the VDH 2.14 motif and the presence of a W (i.e. A or T) in aptamer clone 4-9.

N50 RNA Pool, 97 nt:<br>5′GGGUUUACCUAGGUGUAGAUGCU-N50-AAGUGACGUCUGAACUGCUUCGAA3′
N50 Pool Forward Primer, "42.N50.F", 42 nt:<br>5′GATAATACGACTCACTATAGGGTTTACCTAGGTGTAGATGCT3′
N50 Pool Reverse Primer, "24.N50.R", 24 nt:<br>5′TTCGAAGCAGTTCAGACGTCACTT3′
Motif VDH 2.14, 14 nt: GAACUCAACAUAAR
Aptamer Clone 4-3, 97 nt, 5 nM Kd:<br>5′GGGUUUACCUAGGUGUAGAUGCU**GUAUAUAGCGAACUCAACAUAAGGUAUAAUUACAA UUUCUAUACUUCUUC**AAGUGACGUCUGAACUGCUUCGAA3′
Aptamer Clone 4-9, 97 nt, 9.4 nM Kd:<br>5′GGGUUUACCUAGGUGUAGAUGCU**UCWAUUGAUAUGUUAUAACUGAACUCAACAUAAG GAUAUGAUGUAUGAUC**AAGUGACGUCUGAACUGCUUCGAA3′
Aptamer Clone 3-6, 97 nt, 10.8 nM Kd:<br>5′GGGUUUACCUAGGUGUAGAUGCU**CUGCCCUUCAGAUUUAUCGAUGACCGUUGAACUCA ACAUAAGACCUUCCA**AAGUGACGUCUGAACUGCUUCGAA3′
Minimized Aptamer Variant 3.1, 55 nt, 6.7 nM Kd:<br>5′GGGUAGAUGCUGUAUAUAGCGAACUCAACAUAAGGUAUAAUUACAAUUUCUACCC3′
